# Lysosomes and Their Role in Regulating the Metabolism of Hematopoietic Stem Cells

**DOI:** 10.3390/biology11101410

**Published:** 2022-09-27

**Authors:** Tasleem Arif

**Affiliations:** Department of Cell, Developmental & Regenerative Biology, Icahn School of Medicine at Mount Sinai, New York, NY 10029, USA; tasleem.arif@mssm.edu; Tel.: +1-212-241-4143

**Keywords:** lysosomes, mitochondria, HSCs, glycolysis, metabolism, quiescence

## Abstract

**Simple Summary:**

Hematopoietic stem cells (HSCs) are an essential component of the cellular architecture of the body. These cells are responsible for self-renewal as well as the production of hematopoietic progenitors and adult blood cells. HSCs have been successfully used as a treatment for a variety of hematological disorders, including lymphoma, leukemia, anemia, and anomalies in the immune system. However, owing to the difficulties associated with the limited supply of HSCs, the majority of hematological conditions cannot be treated in clinical settings. It is possible that understanding the intrinsic and extrinsic regulators of HSC stemness could pave the way for developing novel methods for accessing alternative HSC sources. This would enable researchers to get around the limitations that currently exist in clinical applications.

**Abstract:**

Hematopoietic stem cells (HSCs) have the capacity to renew blood cells at all stages of life and are largely quiescent at a steady state. It is essential to understand the processes that govern quiescence in HSCs to enhance bone marrow transplantation. It is hypothesized that in their quiescent state, HSCs primarily use glycolysis for energy production rather than mitochondrial oxidative phosphorylation (OXPHOS). In addition, the HSC switch from quiescence to activation occurs along a continuous developmental path that is driven by metabolism. Specifying the metabolic regulation pathway of HSC quiescence will provide insights into HSC homeostasis for therapeutic application. Therefore, understanding the metabolic demands of HSCs at a steady state is key to developing innovative hematological therapeutics. Lysosomes are the major degradative organelle in eukaryotic cells. Catabolic, anabolic, and lysosomal function abnormalities are connected to an expanding list of diseases. In recent years, lysosomes have emerged as control centers of cellular metabolism, particularly in HSC quiescence, and essential regulators of cell signaling have been found on the lysosomal membrane. In addition to autophagic processes, lysosomal activities have been shown to be crucial in sustaining quiescence by restricting HSCs access to a nutritional reserve essential for their activation into the cell cycle. Lysosomal activity may preserve HSC quiescence by altering glycolysis-mitochondrial biogenesis. The understanding of HSC metabolism has significantly expanded over the decade, revealing previously unknown requirements of HSCs in both their dividing (active) and quiescent states. Therefore, understanding the role of lysosomes in HSCs will allow for the development of innovative treatment methods based on HSCs to fight clonal hematopoiesis and HSC aging.

## 1. Introduction

It is thought that the quiescent state of hematopoietic stem cells (HSCs), like that of most adult stem cells, protects them from the replicative and metabolic stress that would otherwise shorten their lifespan. So, HSC potency, which is the functional capacity of HSCs to promote multilineage engraftment for a protracted duration in lethally irradiated recipient mice, is inexplicably linked to quiescence. HSCs play a pivotal role in the development of all sorts of blood cells [1]. The blood system relies on these multipotent cells, which can differentiate into many types of adult hematopoietic lineages, whether the body is resting or under stress [2]. Most HSCs maintain a dormant state to avoid depletion caused by replication stress [3]. HSCs divide frequently to maintain their stem cell potential, producing progenitors and quiescent daughter HSCs [4]. The metabolic requirements of HSCs are crucial for our understanding of HSC biology both when they are quiescent as well as in an active state. Quiescent/dormant HSCs showed low metabolic activity, limited protein synthesis, and reduced mitochondrial activity [5,6,7,8,9]. High levels of hypoxia-inducible factor (Hif-1) expression and the predominant use of glycolysis for energy supply are characteristic of HSCs [10]. The oxidation of fatty acids is also essential for HSC homeostasis [11,12]. The release of HSCs from quiescence is accompanied by increased mitochondrial activity, reactive oxygen species (ROS) production, and protein synthesis [8,13,14,15]. A metabolic switch between HSC quiescence and the active state requires glycolysis or OXPHOS for HSC metabolism [16].

Lysosomal research acquired substantial attention after the discovery of autophagy, where lysosomes are involved in a degradative cellular process essential for recycling waste materials and preserving cellular homeostasis. However, during autophagy, lysosomes were only seen as a place for cellular waste compartments in cells [17,18]. In recent years lysosomes function as stress sensors, and controllers of cell responses to environmental stimuli such as nutrition and growth hormones have been identified in HSCs [18,19]. Lysosomes are involved in multiple functions; they attracted attention from researchers worldwide as a possible therapeutic target for cancer. More recently, lysosomal role in the maintenance of HSCs has been demonstrated [20,21,22,23].

The aging process is the primary factor in the development of numerous diseases, including immunological deficits and myeloid cancers, and the aging of HSCs is the root cause of many blood disorders. Loss of quiescence with aging is expected to cause HSC deficiencies, as quiescence preserves HSC health and function [24]. A multitude of new information on lysosomes, central to the regulation of HSC metabolism and maintenance, has accumulated rapidly over the past few years, showing that HSCs require a more nuanced metabolic environment than was previously believed to be necessary.

## 2. Quiescence vs. Active State

HSCs are often characterized as either in quiescent or active states [25,26]. Multiple studies have shown that HSCs remain quiescent/dormant and reside in hypoxic bone marrow (BM) niches. Quiescence helps to decrease cellular damage from physiological or oxidative stress and increase self-renewal capacity [26,27]. The molecular structure and function of quiescent HSCs are distinct from those of active HSCs and progenitors. HSCs have a lower mitochondria content and activity of mitochondria compared to progenitors [28,29,30]. Immunophenotypic quiescent HSCs have a lower mitochondrial membrane potential (MMP), lower endogenous nicotinamide adenine dinucleotide (NADH) fluorescence, reduced oxygen consumption, low levels of adenosine triphosphate levels (ATP), and less or no use of glycolysis compared to active HSCs and progenitors [9,29,31].

ROS act as signaling molecules for numerous biological responses required for cellular function, and excessive ROS are harmful to cells, including HSCs [32]. It is known that various mechanisms control the physiological formation of ROS in stem and progenitor cells [33]. Multiple studies have shown that HSC/progenitor cells with a minute increase in ROS are primed for differentiation. In contrast, HSCs with low ROS levels maintain their self-renewal capability [34,35,36,37], and HSCs, with low levels of oxygen, protect HSCs from oxidative stress [26,38].

Various groups have extensively studied the role of ROS on HSC function, and an increase in ROS levels impairs HSC maintenance [33]. Mutant polycomb complex protein BMI-1, also known as polycomb group RING finger protein 4 deficiency, induces mitochondrial dysfunction, decreased ATP production, increased ROS levels, and DNA damage in HSCs [39]. It has been shown that HSC self-renewal requires ATM (ataxia telangiectasia mutated) mediated regulation of oxidative stress, and loss of ATM leads to BM failure, which is coupled with higher ROS [34]. FoxO transcription factors promote antioxidant enzyme synthesis and protect HSCs from oxidative stress [37]. FoxO knockout HSCs have been shown to have poor self-renewal capacity and a massive increase in ROS levels [37].

In the end, maintaining balanced levels of ROS is essential for sustaining the stem cell pool, both in homeostasis and as well as in stress.

## 3. Factors Regulating Glycolysis in HSCs

HSCs relied mainly on glycolysis and increased OXPHOS as they differentiated. Hif-1α acts as a primary mediator for maintaining glycolytic levels in HSCs by regulating the transcription factors of multiple downstream targets involved in glycolysis. The HSCs appear to express Hif-1α even under normoxic conditions, despite the fact that Hif-1α can interact with Hif-1α to create a heterodimer and is relatively stable under hypoxic conditions [40]. It has been shown that several signaling pathways are essential for maintaining Hif-1α stability. For example, the von Hippel–Lindau (VHL)-mediated deubiquitination pathway is required to maintain Hif-1α levels and stability [41,42]. 5’AMP-activated protein kinase (AMPK)- or NAD-dependent deacetylase sirtuin-1 (SIRT1)-mediated signaling is also involved in the maintenance of Hif-1α levels and stability [43]. AhR/aryl complex modulates Hif-1α protein levels which inhibits the interaction between the aryl hydrocarbon receptor nuclear translocator (ARNT) and Hif-1α. Moreover, AhR antagonists are responsible for the increased in vivo growth of HSCs [44]. AhR antagonists induce ROS stress; thus, Ahr molecules may help to maintain HSC functions.

A member of the HOX family of DNA-binding transcription factor, myeloid ecotropic viral integration site 1 (Meis1) is overexpressed in HSCs [45]. Meis1 deletion promotes embryonic lethality due to severe hematopoiesis defects. Meis1 regulates the glycolytic level and HSCs self-renewal through the Hif-2α/ROS/p16 pathway [9,40,46]. Meis1 null HSCs lose their quiescence, elevate ROS levels, and inhibit the expression of Hif-1 and Hif-2; this effect is reversible when HSCs are treated with a ROS scavenger N-acetylcysteine [47]. Human peripheral blood HSCs have been found to preferentially utilize glycolysis as an energy source through Meis1/Hif-1/Pbx1/HoxA9 pathways, suggesting that HSC cell fates are regulated by intrinsic metabolic networks [40].

HSCs glycolysis and function are regulated by Hif-1α, via multiple downstream targets, such as lactate dehydrogenase A (LDHA), pyruvate Kinase M2 (PKM2), glucose transporter 1 (GLUT1), 6-phosphofructokinase, liver type (PFKL), pyruvate dehydrogenase kinase 2 (PDK2), and teratocarcinoma-derived growth factor 1 (TDGF1). Hif-1α inhibits pyruvate from entering the tricarboxylic acid cycle (TCA), thus inhibiting OXPHOS levels [48]. Deleting the Hif-1α downstream targets such as LDHA or PKM2 decreases HSC function and repopulation capacity [48]. Quiescent HSCs display high levels of Hif-1α, which is also relatively stable as the cells migrate from the hypoxic BM niche into the circulating blood [40,49]. Hif-1α deletion in adult HSCs reduces reconstitution capacity and loses quiescence, indicating Hif-1α is needed to maintain the stem cell pool [10]. Furthermore, alterations in the vascular endothelial growth factor caused by mutations in the Hif-1α impair HSC stem cell activity and function [50]. HSCs switch from glycolysis to mitochondrial respiration when Hif-1α is conditionally deleted [47]. Nevertheless, it is also shown that deletion of Hif-1α has no effect on HSC activities [51], but these studies cannot entirely exclude the idea that Hif-1α may play a vital role in BM niche cells to support hematopoiesis.

Moreover, Hif-1α also interacts with Notch and Wnt signaling to control the cell fates of HSCs. Hif-1α interacts directly with Notch’s intracellular domain (NICD), which retains Notch stability and activates downstream targets [52]. Interaction of Hif-1α and catenin promotes the expression of genes influenced by Hif-1α, but it decreases the expression of genes influenced by Wnt signaling [53].

As shown above, Hif-1α seems to have a critical function in hematopoiesis at various stages of development. Deletion of Hif-1α in mice is embryonically fatal due to many abnormalities in vascular development, blood cell development, and nerve cell development [54]. Mice lacking Hif-1α have a much smaller yolk sac and fewer hematopoietic cells compared to wild-type mice [55]. Several lines of evidence demonstrate the role of glycolysis in HSCs, although the underlying regulatory networks or mechanisms still need more investigation. However, the idea of quiescent HSCs using glycolysis as their energy source has been recently challenged [22]. This review summarizes a brief overview of some recent findings in the fields of study that contradict the traditional view of glycolysis in HSCs.

## 4. Glycolysis Is Required Mainly by Activated HSCs

The molecular basis that allows stem cells to store and release nutrients to generate energy in response to different physiological environments has not yet been elucidated [56,57]. Additionally, it has recently been argued that glycolysis is not the primary energy source for quiescent HSCs [22]. Extensive work over the past decade has shown that quiescent HSCs have low metabolic activity, low protein synthesis, low mitochondrial membrane potential, low oxygen consumption, and reduced levels of ATP production [8,9,10,13,14,15,22,58]. HSCs depend primarily on anaerobic glycolysis, as shown by increased glycolytic flux and enzyme expression [10]. During anaerobic glycolysis, glucose-derived pyruvate is converted to lactate by the enzyme LDHA.

Moreover, pyruvate is transported to the mitochondria and converted to acetyl coenzyme A (acetyl-CoA) by pyruvate dehydrogenase (PDH) to activate the TCA cycle. It has been reported that TCA cycle metabolites, such as 2-oxoglutarate, acetyl-CoA, and succinyl-CoA, were absent, whereas glycolysis-related metabolites were found in HSCs [48]. HSCs overexpress pyruvate dehydrogenase kinases (Pdks1–4) relative to progenitors and differentiated cells. It has been reported that Pdk2-/- and Pdk4-/- reduce HSC function and quiescence [48]. These results indicate that glycolysis plays a critical role in maintaining HSC quiescence.

**Figure 1:**
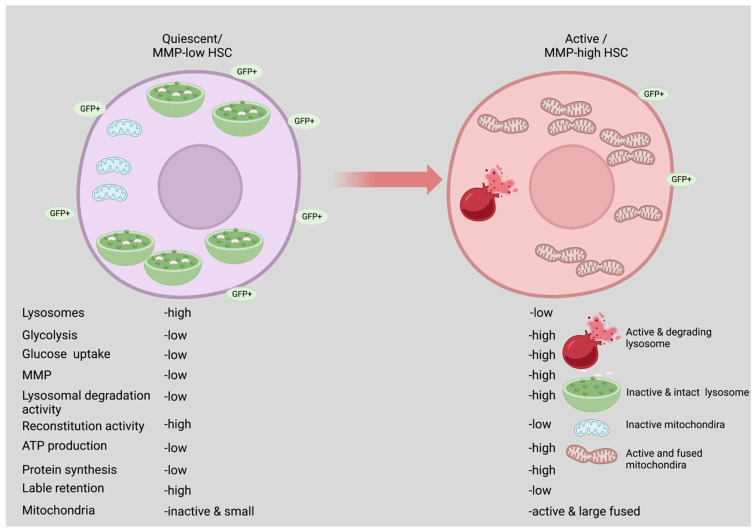
**Quiescent vs. activated HSCs.** Quiescent HSCs with low mitochondrial membrane potential are very potent as compared to high mitochondrial membrane potential (MMP). Quiescent HSCs with low MMP have inactive lysosomes and more in number and size. Activated HSCs (MMP-high) have more lysosomal activity and degrading potential, glycolysis, and active and fused mitochondria. Created with BioRender.com 21 September 2022.

However, a study published in 2020 by Liang, Arif, and colleagues [22] challenged the above concept and showed that glycolysis is used mainly by activated HSCs as a source of energy. They established that subpopulations of HSCs with a low mitochondrial membrane potential (MMP-low) are nonetheless functional [9,58]. MMP-low HSCs are more quiescent and exhibit lower transcription and translation compared to HSCs with high mitochondrial membrane potential (MMP-high). Liang, Arif, et al. further showed that the majority of label-retaining green fluorescent protein positive (GFP+) HSCs were within MMP-low HSCs compared to MMP-high HSCs [22] (Figure 1). Suggesting that HSCs maintain label-retaining proprieties until they undergo division, MMP-low HSCs are specifically in the G_0_ phase of the cell cycle. Interestingly, EPCR, an immunophenotypic marker of self-renewing HSCs, was expressed higher in MMP-low HSCs [59]. MMP-high HSCs, on the other hand, are in an active HSC state, as more than 55% of MMP-high HSCs are in the G_1_/G_2_/S/M stages of the cell cycle [22]. MMP-high HSCs showed enhanced oxygen consumption, NADH levels, and ATP content are all consistent with decreased repopulation activity. In MMP-high HSCs, anabolic pathways that promote transcription, translation, and cell cycle progression are highly elevated [22]. Unlike quiescent MMP-low HSCs, active MMP-high HSCs expressed more glycolytic enzymes such as GLUT1/ solute carrier family 2 (SCL2A1), GLUT4/SLC2A2, Hexokinase 1 (HK1), and LDHA. In addition, 2-deoxyglucose inhibited glycolysis in MMP-high HSCs but not in MMP-low HSCs in the petri plate. These results indicate that MMP-high HSCs, but not MMP-low HSCs, rely heavily on glycolysis for survival. Furthermore, inhibition of glycolysis in MMP-high HSCs significantly enhanced over 70-fold the regenerative capacity, with limited or no effect on MMP-low HSCs [22] (Figure 1).

These results show that non-glycolytic metabolic pathways are very important for HSC homeostasis and quiescence.

## 5. Lysosomal Metabolism

In eukaryotic cells, lysosomes are membrane-bound organelles with an acidic lumen that were first identified in 1955 by Christian de Duve [60,61], and act as degradative organelles, and cellular metabolism regulators. Lysosomes degrade damaged mitochondria, pathogens, lipids, proteins, carbohydrates, and DNA/RNA molecules which are transported to lysosomes by several pathways and converted into a wide variety of molecules and nutrients that are reused by the cells [62]. Autophagosomes transport intracellular substrates, while phagosomes and endosomes transport extracellular material to lysosomes [63]. In addition, chaperone-mediated autophagy (CMA) an independent process, helps transport proteins across the lysosomal membrane. More than sixty lysosomal hydrolases degrade lysosomal cargo before being shuttled to the cytosol for further catabolic/anabolic processing. Lysosomes have been studied for their degradative function since their discovery [61], recent research is shifting the focus away from them as “unregulated cellular waste bags” toward their role as highly dynamic organelles at the hub of cellular metabolism [62]. Lysosomes were found to play a critical role in metabolic signaling, gene regulation, plasma membrane repair, antigen presentation, cell adhesion, apoptosis, pro-inflammatory response, and exocytosis [64,65,66,67,68,69], and more recently, lysosomes were found to regulate the maintenance of HSCs [22].

Lysosomes interact with other cellular organelles by multiple ways (Fusion and non-fusion interaction). Lysosomal membranes are repaired, and antigen presentation is performed by fusion during autophagosomes formation [70,71,72]. Lysosomes are known to interact with the endoplasmic reticulum (ER), peroxisomes, and mitochondria, and transport cholesterol [73]. Regulation of mTOR complex 1 (mTORC1) activity and localization of lysosomes around the nucleus requires interaction with the Golgi bodies [74]. Multiple studies have shown that interactions with mitochondria influenced lysosomal acidity and played a role in the regulation of mitochondrial fission [75,76].

The activity of critical players, such as mTORC1, 5-AMP-activated protein kinase (AMPK), or glycogen synthase kinase-3 (GSK3b) in cellular metabolism is modulated by lysosomes [77,78,79], and activated when translocase to the lysosomal surface [80].

Lysosomal regulation influences development, differentiation, and a feedback loop that relates lysosomal biogenesis to nutrient availability [68]. Transcription factor EB (TFEB), a master regulator of lysosomal biogenesis, is phosphorylated by mTORC1 in response to a lysosomal amino acid (AA), translocating to the nucleus and enhancing transcriptional activity of lysosomal, and autophagy genes [80]. Lysosomal genes are regulated by several transcription factors in the MiTF family, including melanocyte inducing transcription factor (MITF), transcription factor E3 (TFE3), and transcription factor EC (TFEC) [18,81]. Other transcription factors, such as zinc finger with KRAB and SCAN domains 3 (ZKSCAN3), and bromodomain-containing protein 4 (BRD4), as well as the Foxo family, modulate the expression of lysosomal proteins and biogenesis [82,83,84,85].

Thus, lysosomes are responsible for regulating a variety of cellular functions, and changes or modulations to lysosomes can impact key physiological pathways.

### Lysosomal Disorders

During lysosomal dysfunction, the significance of lysosomes in cellular metabolism is very important. Lysosomal storage disorders (LSDs) are a collection of seventy rare and severe genetic diseases caused by mutations in lysosomal hydrolases that interfere with their enzymatic activity and stability [23]. The buildup of substrates in lysosomes is a hallmark pathogenic feature of LSDs, which frequently leads to inhibition of other hydrolases and accumulation and limiting lysosomal functions. A lysosomes failure to degrade cargo leads to disruption in autophagy or mitophagy, vesicle trafficking, fusion with other organelles, mitochondrial function, and signaling cascades. LSDs usually cause people to become disabled and die early age [23,86].

Lysosome-associated proteins or genes in neurodegenerative disorders, including Alzheimer’s, Huntington’s, and Parkinson’s, are altered, and mutations in lysosomal proteins constitute the most important known risk factor [63,87]. Further, defects in lysosomal signaling have been linked to cancer and aging [88].

Most cancer cells have altered metabolism [89,90,91]. Cancer patients often have mutations in several metabolic pathway components, allowing these pathways a potential therapeutic target. Targeting lysosomes may help to combat cancer, eliminate leukemia stem cells (LSCs), and chemoresistance [21,92]. Targeting lysosomes in LSCs may improve comprehensive response, recurrence, and duration when coupled with chemotherapy or HSC transplantation. Schofield (1978) introduced the idea of a physiological niche of stem cells in the BM [93]. Recent findings have broadened our knowledge of the metabolic and lysosomal components essential for the functional maintenance of HSCs [22].

Leukemia is caused when HSCs undergo mutations or epigenetic alterations [94]. LSCs originate as disease progress, chronicity or acuteness of leukemia, and myeloid or lymphoid immunophenotype. HSCs sustain lifelong self-renewal capacity; thus, HSCs have a higher frequency of mutations than less primitive cells [95]. Leukemic cells develop from progenitors with mutations that enable self-renewal, and genetic and epigenetic changes in HSCs reduce cell death and enhance self-renewal [96].

Very few lysosome-targeted therapies are currently in the clinical use [97]. Therefore, identifying individuals with genetic history most likely to react to a drug could be a major advance in the field. Epigenetic variables that can distinguish lysosome-directed therapy responders and inherent lysosomal malfunctions are not known. Further research is needed to acquire more degree of understanding, which depends on disease nature, variability, and prevalence.

Recent research demonstrates that higher lysosomes and inhibited lysosomal activity/degradation capacity are critical for the regulation of quiescent HSCs [21]. Thus, lysosomes may regulate HSC maintenance and serve as a therapeutic target in hematological diseases.

## 6. Role of Lysosomes in Maintaining HSCs Quiescence

**Figure 2:**
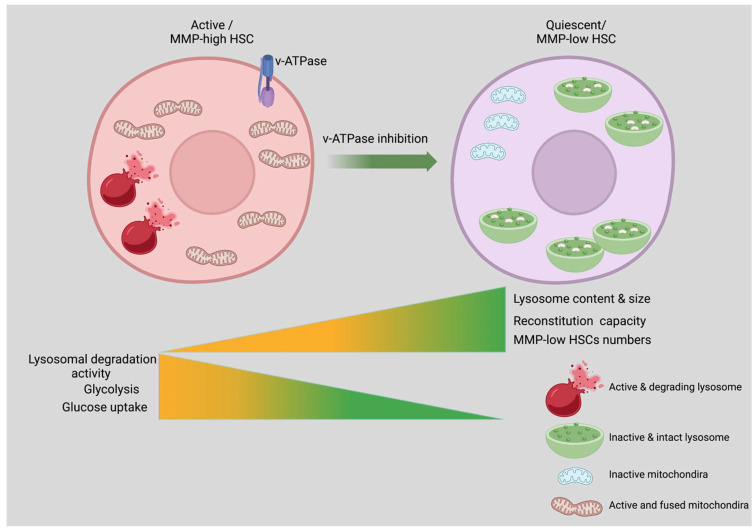
**Lysosomal repression inhibits the activation of HSCs.** Lysosomal turnover is reduced by ineffective lysosomal activity, which results in the accumulation of nutrients, including amino acids. Nutrients that are released from lysosomes maintain HSCs dormant and potency. The stem cell potency of activated HSCs is enhanced or improved by v-ATPase inhibition. Created with BioRender.com 21 September 2022.

Nearly all eukaryotic cells rely on lysosomes as their primary degradative organelle. They help maintain cellular balance, and defects in lysosomal function have been linked to several diseases [21]. Major regulators of cellular signaling have been shown to be activated at the lysosomal surface [21], and in recent years, lysosomes have become the control hubs of cellular metabolism. Recent research demonstrates that quiescent HSCs have an overabundance of large lysosomes, which is commensurate with the mRNA expression of genes encoding lysosomes and protein degradation pathways [22]. Lysosomes are known to be involved in endocytosis, phagocytosis, and autophagy. However, lysosomes do more than just degrade cargo; they also serve as storage sites for metabolites and nutrients such as amino acids (AA). The byproducts secreted from the lysosome are reused for energy regulation [98]. Quiescent MMP-low HSCs have a greater number of lysosomes and a lower pH than activated MMP-high HSCs. Autolysosomes are present in greater numbers in the MMP-low HSCs, and mitochondria are sequestered into lysosomes (Figure 1), suggesting that they may initiate mitophagy, a process of mitochondrial clearance. In addition, MMP-low HSCs had higher expression of the TFEB and inhibited lysosomal degradative activity [22]. However, suppression of lysosomal activation enhanced quiescence and improved the competitive repopulation capacity of MMP-low and -high HSCs up to 9- and 90-folds, respectively (Figure 2) [22].

In the past, lysosomes have been linked to cellular dormancy. Even though autophagy flux decreased during prolonged quiescence in rat embryonic fibroblasts or neural stem cells, lysosomal gene expression was shown to be elevated [99,100]. The deeper the cells went into quiescence in response to a decrease in lysosomal function, the more activated they became in response to an increase in function [99,100]. From these results, we can infer that lysosomal cargo sequestration is critical for maintaining cellular quiescence, while cargo breakdown and release of nutrients are critical for triggering HSC activation. Human HSCs (huHSCs) have also been shown to illustrate the role of lysosomes in HSC activities. Increased expression of TFEB and the autophagy-lysosomal pathway was observed in quiescent huHSC [101]. HSC repopulation activity was maintained while TFEB overexpression induced quiescence. Lysosomes in human HSCs actively break down receptors, such as the transferrin receptor CD71, to inhibit cell activity and entry into the cell cycle [101]. Interestingly, huHSC and muHSC have different lysosomal activity requirements.

**Figure 3:**
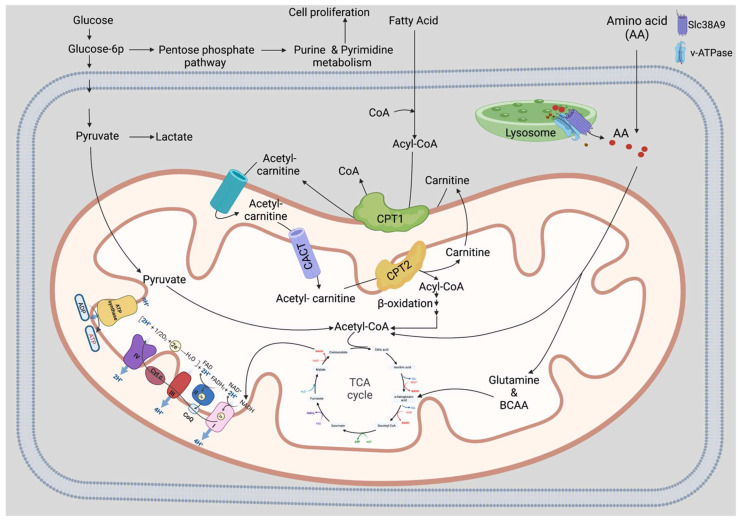
**Role of mitochondria and lysosomes in HSC maintenance.** The metabolic mechanisms that aid in HSC self-renewal, differentiation, and glycolysis fuel the metabolic pathway for active HSCs. In the glycolysis pathway, glucose is degraded into glucose-6P, which undergoes a series of reactions and is converted to pyruvate. Pyruvate generates energy for active HSCs in the TCA cycle. Simultaneously, the production of nucleotides like purine and pyrimidine also requires glucose-6P and utilizes in the pentose phosphate pathway, which is critical for cell proliferation and activating HSCs adapted with permission [6]. Alternatively, HSCs are activated by unknown mechanisms byproducts such as amino acids (AAs), or other nutrients release from the lysosomes may be shuttled to the mitochondria from the cytosol, where they are converted to acetyl-CoA and enter the TCA cycle to produce ATP. HSCs are activated when TCA cycle is activated by alpha-ketoglutarate (α-KG), the end product of glutamine in the mitochondria. Instead of using glucose for energy generation, the catabolism of branched-chain amino acids (BCAAs) also feeds the TCA cycle through α-KG to produce ATP. FAO, a crucial ATP-producing catabolic pathway, feeds the TCA cycle rather than glycolysis. Long-chain fatty acids are converted into short chain fatty acyl-CoA in the cytosol and subsequently transported by carnitine palmitoyltransferase 1 (CPT1), carnitine-acylcarnitine translocase (CACT), and carnitine palmitoyltransferase (CPT2) into the mitochondria through carnitine pathway. Here, β-oxidation yields acetyl-CoA to drive the TCA cycle adapted with permission [6]. FAO is vital to HSC maintenance. Created with BioRender.com 21 September 2022.

Notably, quiescent neural stem cells (NSCs) were found to have more and bigger lysosomes than active NSCs [100]. Insoluble protein concentrations in large lysosomes impeded stem cell activation in aged quiescent NSCs, while increasing their lysosomal activity, thus, reinstating a younger state [100]. It is unknown how lysosomes influence quiescence in other adult stem cell types. Nutrients such as AAs released during lysosomal degradation may be essential for activating HSCs by unknown mechanisms (Figure 3). Lysosomes may be able to manage the level of quiescence and the rate of activation of HSCs. Carefully monitoring the degradation rate, and the transport of metabolites and AAs to the cell will increase our knowledge of maintaining HSCs activity and function. Therefore, controlling lysosomal degradation might be used to convert active HSCs back into quiescent HSCs, which may be useful for clinical purposes, such as for BM transplantations or targeting hematological disorders.

### 6.1. Glycolysis and Mitochondrial Biogenesis

Multiple studies have reported increased MMP and metabolic activity in HSC, pushing them into the cell cycle [12,13,14,15,22]. A fused mitochondrial network and expanded crista activate mitochondria to support increased surface area for electron transport enzymes [22]. Interestingly, the expression of genes encoding both glycolysis and mitochondrial activity increases in the hematopoietic stem and progenitor cells (HSPCs) and activated HSCs [12,13,14,15,22]. Furthermore, activated HSCs rely on glycolysis [22]. Glucose uptake also increases when the TCA cycle is triggered by dimethyl α-ketoglutarate in MMP-low HSC [22]. Notably, it is known that the activity of the mitochondrial electron transport chain is necessary to keep HSCs in a quiescence state [102,103].

Progenitors or highly differentiated HSCs showed a high glucose uptake, while HSCs in the BM have a reduced glucose uptake [104]. Liang, Arif, et al. showed that pharmacological suppression of lysosomal function by Concanamycin A (ConA) inhibits lysosomal degradation, enhances quiescence in HSCs, and decreases glucose uptake. Suppressing lysosomes enhances the HSCs competitive repopulating capacity and improves their ex vivo maintenance of functional HSCs (Figure 2) [22]. Thus, active HSCs require a higher glucose intake to sustain entry into the cell cycle, while quiescent HSCs maintain their overall low metabolic activity. Hence, low metabolic activity protects MMP-low HSCs from premature exhaustion and ensures their survival, thus maintaining HSCs quiescence [22].

These results suggest that non-glycolytic metabolic pathways play a critical role in maintaining HSCs. These data imply that HSC activation is related to glycolysis and mitochondrial biogenesis to enhance ATP production in HSCs.

### 6.2. How HSCs Switch from Catabolic to Anabolic Activity?

HSCs are subjected to a particular metabolic requirement from a low to a high proliferative state during the shift from catabolic to anabolic activity. Genes encoding in both human and murine that enable transcription, and translation have been found to be upregulated in active HSCs [13,14,22,105]. TFEB and cMYC expressions are altered in huHSCs as they exit quiescence. In active HSCs, TFEB is overexpressed, whereas cMYC expression downregulates. Furthermore, inducing stress in HSCs enhances ribosomal and mitochondrial biogenesis [101]. cMYC levels are elevated in huHSC, while lysosomal catabolism is suppressed [101]. Hence, HSC activation is connected with reprogramming from a catabolic to an anabolic state. Lysosomal inheritance was followed using advanced in the aging of HSCs isolated from murine or humans and tracking of single HSCs over time to correlate HSC fate [106,107]. Using a fluorescent reporter of NUMB endocytic adaptor protein (NUMB) is found to be decreased and shows that NUMB inheritance is enhanced in a cell expressing differentiation markers such as B-lymphocyte activation marker (CD48), endoglin (CD105), and transferring receptor-1 (CD71), but not platelet glycoprotein IIb (αIIb; CD41) and stem cells antigen-1 (Sca-1) [108]. CD71 expression was found to be highly correlated with other markers of HSC activation, including mitochondrial activity, ROS generation, and cMYC overexpression. In another study using a lysosomal marker lysosomal-associated membrane protein 1 (LAMP1)- Venus reporter, a pH-sensitive fluorescent lysosome showed a low number of lysosomes in the cells [107].

It is still unclear how the release of nutrients from lysosomes activates HSCs [22]; this may be due to HSCs requiring high lysosomal catabolic activity to exist quiescence. Lysosomal degradation releases several nutrients and intermediate metabolites, which are used in the mTORC1 pathway to activate HSCs. It is well known that lysosomes have been linked to a wide variety of autophagy pathways. HSCs in adult mice are maintained by CMA, a selective form of the lysosomal degradation pathway [109]. In differentiated HSCs, CMA is overexpressed, and it plays an important role in maintaining HSCs, proteins involved in the synthesis of linoleic acid metabolism, fatty acid (FAs) biosynthesis, and oxidation. Moreover, CMA is not required for mitochondrial quality control. Fatty acid desaturase 2 (FADS2) inhibitors affect HSC functions, whereas linolenic acid, a direct product of FADS2, has been shown to rescue CMA-deficient HSC functions in vitro and in vivo [109].

Several studies reveal that HSCs retain mitochondria in a quiescent state, while mitochondrial structure and functions are altered during HSC activation or oxidative stress [13,22,103,110]. HSCs metabolic switch from catabolic to anabolic state is an intriguing phenomenon, but how it occurs remains an open question. It is still unclear how HSCs lysosomal degradation, and release of cargo activate HSCs. It is unknown how altering lysosomal activity plays a role in maintaining HSC quiescence.

### 6.3. Role of Anabolic Regulators in HSCs

A decrease in protein synthesis is an important and unique characteristic of quiescent HSCs [8,111]. Hence suppressing anabolic activity using pharmacological molecules may promote HSCs function and activity. mTORC1 is one of two protein complexes containing the essential kinase mTOR [88] and plays an important role in regulating protein synthesis in cells, including HSCs and inhibiting phosphorylation of S6 and 4EBP1 [112] altered protein synthesis in HSCs [113]. HSCs depend heavily on the control of mTORC1 activity. Depletion of S6 and 4EBP in HSCs coincides with cell cycle progression, and mitochondrial activation after mTORC1 is hyperactivated by knocking out tuberous sclerosis complex 1 (TSC1) or overexpressing Ras homolog enriched in brain (Rheb) [114]. Phosphatase and tensin homolog (Pten), a negative regulator of the PI3K-AKT pathway, is essential for inhibiting mTORC1 signaling in quiescent HSCs. Deleting Pten altered HSC numbers due to activation of mTORC1, whereas overexpression of p16^Ink4A^ and p53 induces abnormal cell cycle progression in HSCs [115]. Rapamycin, an mTOR inhibitor, enhances HSC function, activity, and reconstitution capacity, and further increased mTORC1 activity induces HSC failure in vivo. Similarly, the reconstitution capability of HSCs is diminished when Raptor is knocked down in HSCs, which activates mTORC1 [116].

Liang, Arif, and colleagues show that MMP-high HSCs contain lower lysosomal content, which is linked to mTOR expression, recruitment, and activation [22]. It is well known that gene translation and cell proliferation require mTOR activation [117]. Therefore, activating mTOR signaling enhanced downstream phosphorylation of mTORC1 target 4EBP1 and upstream targets (Rheb1 and RAGA/B). MMP-low HSCs express higher TFEB levels, a regulator of lysosomal biogenesis that inhibits mTORC1. Interestingly, mTOR expression and activity were significantly reduced in MMP-low HSCs and may be promoting HSCs quiescence [22]. Lysosomal suppression by using ConA lowered mTOR protein levels, its upstream targets (Rheb1 and RAGA/B), as well as its downstream target (4EBP1) in MMP-high HSCs [22]. Therefore, lysosomal suppression by ConA increases quiescence in HSCs by reducing the expression levels of Ki67, a marker of cell proliferation, and CDK6, which predicts HSC exit from G_0_, both of which are associated with active MMP-high HSCs [22].

In sum, mTORC1 is essential for maintaining HSCs function and activity, whereas high levels of mTORC1 activity induce significant abnormalities in HSCs function and activity. Raptor, Rheb1 also activates mTORC1, and knocking down Rheb1 inhibits mTORC1 activity [118]. Raptor-/- vs. Rheb1-/- HSCs showed a distinct difference in competitive reconstitution assay in vivo [118], while deleting Raptor or Rheb1 increases the total number of HSCs, inhibits function, and reduces HSC competitiveness in vivo, deleting Raptor, but not Rheb1, expands HSCs, and decreases stem cell capacity while expanding HSCs without Rheb1 retain their stemness. These results indicate that maintaining HSC self-renewal activity in vivo moderate’s levels of mTORC1 activation [119,120]. Nutrient-sensing may play a critical role in the maintenance of HSCs in culture, whereas HSCs treated with rapamycin and GSK-3 inhibitor enhance HSCs number and function both in vitro and in vivo [121]. Rapamycin-treated HSCs ameliorates aged HSCs [122]. Hence maintaining or increasing healthy HSC populations in vitro and in vivo requires strict control of mTORC1 activity.

Therefore, this review highlights metabolic regulatory factors that are important to HSC biology.

## 7. Nutrients in the Maintenance of HSCs

The degradation of macronutrients, such as carbohydrates, lipids, proteins, vitamins, and minerals, into smaller molecules, allows living organisms to use these molecules in anabolic and catabolic metabolic pathways. In order to maintain HSCs quiescence and functional capabilities, anabolic and catabolic reactions must work together (Figure 3). Several critical nutrients, including AAs, lipids, and intermediate metabolites that are important for HSC maintenance, are addressed below.

### 7.1. Amino Acids (AAs)

HSCs have the potential to differentiate into various blood cells and are often considered to be a type of multipotent stem cells which are essential for maintaining the health of blood cells. More than sixty years ago, Harper [123] proposed that AAs imbalance or alteration could be a disease mechanism. Previous studies of HSC maintenance mainly focused on the quiescent vs. the active state, and accumulated evidence indicates that AAs play a critical role in maintaining stem cells activity and function, whereas free AAs are enriched in primitive stem cells by several folds [124]. Three specific AAs, such as valine, methionine, and threonine, are vital for the maintenance of HSC quiescence. Moreover, deprivation of AAs in vitro impaired stem cell repopulation ability. These results show AAs importance in HSC metabolism [125] (Figure 3).

Lower protein translation rates in adult HSCs [8,126] were linked to HSC quiescence compared to progenitors, and multiple AAs emerged as key regulators of HSC function. Valine has been shown to be critical for ex vivo proliferation of both muHSC and huHSC [124]. Moreover, glutamine uptake was found to be critical for HSPCs differentiation into erythroid lineage [127]. In contrast, a diet lacking glutamine led to the inhibition of myeloid leukemias [127,128]. Leucine, an essential AA, has been shown to regulate the mTOR pathway in HSCs [129]. Branch chain amino acids (BCAAs) such as isoleucine and valine contribute to HSC cell proliferation and increase mTOR activity, thus decreasing HSCs function and activity [130]. Moreover, nutrient availability regulates stress pathways and promotes the survival of HSPCs [131].

Alteration of AAs metabolism severely impedes HSC proliferation and survival in vivo. Wilkinson et al. showed that the correct quantity of BCAAs helps maintain HSC functions and survival [132]. Interestingly, an imbalance of BCAAs had a greater impact on HSC proliferation and HSC survival; therefore, alteration in AAs level may play a pivotal role in promoting HSC dysfunction with some unknown mechanism [124,132]. As previously reported, the glutamate transporter (ASCT2) is overexpressed in HSCs. ASCT2 and activation of glutamine metabolism play an important role in the differentiation and reducing stem cell activity of HSCs [127].

Notably, HSCs express Src homology region 2 domain-containing phosphatase-1 (SHP-1), which belongs to a family of protein tyrosine phosphatases called Src homology region 2 domain-containing phosphatase-2 (SHP-2) that regulates intracellular tyrosine phosphorylation levels via the SH2 domain. The ability of HSCs to self-renew is impaired in the absence of SHP-1 [133]. Currently, HSCs are transplanted into immunodeficient mice to examine their self-renewal and differentiation potentials; however, this technique has a remarkably abysmal success rate [134].

Further research into AAs metabolism in HSCs may one day be able to improve the outcome of BM transplants by delivering patients with more nutrients. A recent study demonstrates that AA catabolism controls the GCN2-eIF2α axis in HSCs to modulate protein synthesis. In addition, the NAD+ precursor nicotinamide riboside increases AAs catabolism and activates the GCN2-eIF2α axis in order to maintain the long-term function of HSCs [135]. Finally, AAs metabolism may control how leukemia advance, and targeting AA in HSCs may be a way to treat leukemia [136,137].

The most common use of HSCs nowadays is in BM transplantation, where immune rejection and metabolic abnormalities are important obstacles in the failures of BM transplantation. Thus, targeting AA effectively in HSCs may be used to treat leukemia. More research is needed to determine how release of AAs from lysosomes activates HSCs.

### 7.2. Lipids

Lipids, for example, cholesterol and fatty acid, are critical for HSC self-renewal, asymmetric differentiation, and commitment as fuel and membrane-building macromolecules. Lipid biosynthesis and import pathways are activated in HSCs [138] (Figure 3).

Extracellular receptors that impact HSC proliferation and commitment are found in lipid membranes rich in cholesterol. Alteration in cholesterol transport affects HSC function and activity. Apolipoprotein E (ApoE) deficiency modulates cholesterol efflux, inducing cholesterol to accumulate in the lipid membrane [139]. Lipoprotein uptake leads to a decreased number of hematopoietic precursors such as myeloid, erythroid, megakaryocytic, and lymphoid in the BM, whereas HSC commitment and function is impeded due to the accumulation of cholesterol [140]. Lysosomal acid lipase is the key enzyme that processes lipoproteins to cleave esterified cholesterol and triglycerides to generate free cholesterol and fatty acids (FAs) [141], and its deficiency results in cholesterol deposition in the lysosome, thus enhancing HSCs numbers, common myeloid progenitors (CMPS), and granulocyte-monocyte progenitors (GMPs) [142]. Therefore, lipids alteration in HSCs affects cellular metabolism. A more profound understanding of these facets will shed light on novel approaches that can be used to improve the functionality of HSCs as well as their maintenance.

Lipids serve as a source of energy for fatty acid oxidation (FAO); therefore, fatty acid metabolism significantly impacts HSC functions and activity. In HSCs, Angiopoietin-like 4 (Angptl4) loss increases myeloid lineage production [143,144]. FAs also serve as building blocks for various macromolecules, such as sphingolipids and phospholipids, which are involved in activating hHSCs. Several transcription factors such as hematopoietic transcription factor PU.1, and GATA-binding factors 1 & 2 are activated by sphingolipids, leading to the reprogramming of erythroid-primed multipotent progenitors (MPPs) into myeloid lineages [145]. Sphingosine-1-phosphate (S1P) is reported to play a significant role in the mobilization and the homing of HSC to spleen peripheral regions [145]. It has been found that the production of prostaglandin E2 (PGE2) and hydroxyeicosatetraenoic acid (HETE) by cyclooxygenases (COX) and lipoxygenases (LOX), respectively, increase the self-renewal capacity of HSCs [146].

Recently omics assays have shown that FAO is upregulated in HSCs, showing the importance of lipid metabolism in regulating HSC self-renewal and differentiation [11,147,148]. The FAO pathway is an important catabolic pathway for energy production. Long or medium-chain fatty acids from the cytosol are transported into the mitochondria via the carnitine pathway and produce acetyl-CoA (Figure 3). The acetyl-CoA shuttle into the TCA cycle for the synthesis of ATP and activates HSC [149]. Indeed, HSCs lose their reconstitution capacity by knocking down promyelocytic leukemia (Pml)-regulated peroxisome-proliferator activated receptor delta (Ppar-δ) [11,150]. Moreover, enhancing mitophagy and activating the peroxisome proliferator-activated receptor gamma (Ppar-γ) pathway activates HSC [12]. Inhibition of Ppar-γ using pharmacological inhibitors switches the metabolic pathway from oxidative to glycolytic phosphorylation [151]. Moreover, degradation of proteins via CMA-mediated autophagy upregulates FAs metabolism upon HSC activation [109].

Recent research has shown that HSCs uptake free FAs from the BM microenvironment and undergo a metabolic shift toward FAs metabolism in order to meet their metabolic demand, which activates HSCs, whereas HSCs avoid entering the cell cycle if CD36-mediated free FAs uptake is inhibited [152]. More research is required to understand how FAO metabolism is implicated in HSC maintenance. All this evidence shows a link between intracellular lipid metabolic reprogramming and HSCs maintenance. Factors that disrupt systemic and cellular lipid homeostasis have the potential to interfere with the proliferative and differentiation capacities of HSCs, hence altering the passage of hyper-responsive committed cells from the BM into the blood circulation.

### 7.3. Metabolites

Insights into HSC metabolomics by liquid chromatography mass-spec platform have been challenging due to the relative rarity and low numbers of HSCs. A recent HSC metabolism study identified 160 metabolites in HSPCs, including phosphatidylcholine, glycolytic, and lipid intermediates [108]. Schonberger et al. identified metabolites such as those from the TCA cycle and AAs using a low number of HSCs. Further, using higher numbers of HSCs, they detected polar lipids, bile acids, and retinoids [147]. Notably, another group identified many highly abundant lipids metabolites in HSCs [108]. HSCs were shown to have significant levels of retinoic acid (4-oxo-RA), which is a derivative of retinoic acid (RA) [147,153]. It was also shown that the intermediatory metabolites involved in FAs metabolism were abundant in HSCs, whereas TCA cycle and AA metabolism related metabolites were highly exorbitant in MPPs. Both HSCs and MPPs were enriched with retinoids, high H3K4me3, and reduced H3K27me3 levels, which are implicated in RA metabolism. Furthermore, cytochrome P450 family 26 subfamily B member 1 (Cyp26b1), vital for 4-oxo-RA synthesis, maintains HSC activity and functions [147]. These significant findings contribute to a better insight into the metabolism of HSCs and the determination of HSC function and fate.

Levels of aspartate, purine, and asparagine play an essential role in HSC activity and function during hematopoietic regeneration [154]. Untangling cellular metabolism is made more difficult by the fact that metabolic pathways are connected in interdependent sets of chain reactions. Therefore, intermediate metabolites produced in the metabolic pathway have a variety of possible roles in maintaining HSCs.

In contrast to most other cell types, HSCs do not express the aspartate transporter. Aspartate is synthesized in the cells from glutamate and oxaloacetate with the help of the mitochondrial enzyme glutamic-oxaloacetic transaminase 1 (GOT1) and glutamic-oxaloacetic transaminase 2 (GOT2) in the cytosol and converts aspartate to catabolize AAs, proteins, lipids, and nucleotides. It is well-documented that aspartate can activate the TCA cycle [155]. Aspartate and its catabolized byproducts, such as purine and pyrimidine nucleotides, play a critical role in cell proliferation [154]. Activation of HSCs may be caused by an increase in aspartate metabolism [154]. According to the research above, aspartate metabolites play an essential role in maintaining HSCs function and activity.

### 7.4. Other Nutrients

Other essential nutrients, such as vitamins shown to be implicated in the maintenance of the HSCs functions and quiescence. Vitamin A has been shown to maintain HSC quiescence and dormancy, while a diet lacking Vitamin A promotes HSC impairment [153]. Vitamin D is essential for HSC self-renewal in the Zebrafish hematopoietic system, and human cord blood HSPCs [156]. Additionally, the expression of Vitamin D receptor decreases myeloid progenitor differentiation, HSCs, and LSCs numbers [157]. In mouse models of acute myeloid leukemia, Vitamin D receptor agonists and hypomethylating treatments decrease tumor growth and accelerate LSCs elimination [157]. Interestingly, mouse HSCs were found to overexpress Vitamin C, also known as ascorbate, in all downstream hematopoietic progenitors and knocking down Vitamin synthesizing enzyme (Gulo) enhances HSC frequency, function, and reconstitution capacity [158]. More recently, nicotinamide riboside, an analog of vitamin B3, has been shown to maintain HSC functions and enhances self-renewal potential [159,160].

Nevertheless, a great deal of mystery surrounds the mechanisms by which the metabolism of various nutrients exerts control on HSC activity at various stages of development and needs further study.

## 8. Aging HSCs Metabolism

The aging of HSCs is a major contributor to the development of many hematological disorders, such as myeloid malignancies and immunological deficits. HSCs and progenitor cells are distinct because of their ability to differentiate into multipotent, quiescent, and self-renewing cells. However, the phenotype and function of HSCs are asymmetrical, with distinct stem cell characteristics. Adult HSCs are deeply inactive and are quickly triggered and primed during stress. In contrast to primed HSCs, which are thought to play a major role in stress-induced hematopoiesis repair, dormant HSCs are thought to take longer to enter the cell cycle [153]. Analyses using non-transplant models of native hematopoiesis have shown that MPPs expansion is necessary to achieve steady-state hematopoiesis [161,162]. Long-lived progenitors, rather than HSCs, drive hematopoiesis. Different subsets of huHSCs show a preference for specific lineages, which indicates their ability to differentiate into many lineages [163,164]. Stress hematopoiesis, such as inflammation or recovery after myeloablation, is when lineage-biased HSCs are most abundant. It is unclear if the metabolic status of HSCs is linked to the different types of HSPCs (non-biased HSCs, megakaryocyte-biased HSCs, and long-lived progenitors).

ATP is essential for HSCs proliferation and differentiation during stress hematopoiesis. Cytokine signaling is frequently used to convey sudden increases in the requirement for mature hematopoietic cell formation [165]. Loss of DNA binding 1 (Id1) gene inhibitor decreases mitochondrial OXPHOS, further inhibiting HSCs proliferation due to stimulation of cytokines [166]. Therefore, targeting Id1 may enhance HSC survival and activity in BM transplantations, chronic stress, or aging [166]. Notably, thrombopoietin upregulates mitochondrial metabolism and skews adult HSCs toward megakaryocyte-biased differentiation [167]. Recombinant thrombopoietin or romiplostim elevates HSC MMP and megakaryocyte differentiation. MMP-high HSCs skewed megakaryocyte-lineage during competitive BM transplantation [167]. It is likely that the metabolic imbalance has a consequence on HSC aging and requires further investigation.

## 9. HSC Aging

Understanding the role of aging stem cell function is an area of research interest. Aging leads to a steady loss of function in many organs and tissues and is also the biggest risk factor for cancer [168]. The effects of aging on the murine hematopoietic system are similar to those seen in humans. Because of this, mouse models can be used to study the effects of aging on huHSCs. Therefore, eliminating aging HSPCs rejuvenates the hematopoietic compartment in mice [169].

**Figure 4:**
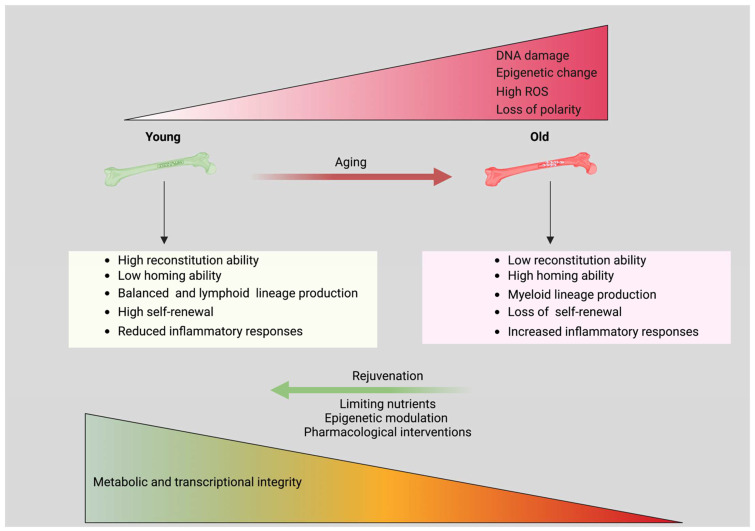
**HSC aging and rejuvenation.** Old HSCs are characterized by a diminished capability for repopulation, impaired homing, increased mobility, myeloid skewing, higher ROS, and enhanced metabolic and transcriptional integrity. DNA damage, ROS, epigenetic alteration, and polarity loss are the main causes of HSC aging. By limiting nutrients and adjusting epigenetic expression, aged HSCs may be revitalized. Created with BioRender.com 21 September 2022.

Hallmarks of aged HSCs are DNA damage, cellular senescence, increased ROS production, increased frequency, a decreased ability to regenerate, and increased output of myeloid cells and mitochondrial dysfunction [170] (Figure 4). With age, autophagy and proteasomal degradation decline, and DNA damage accelerate. HSC function and activity are affected due to the enhanced mitochondria OXPHOS, ROS production, and higher levels of ROS induce HSC dysfunction [171]. HSCs niche structural and functional modification increases myeloid-lineage cell growth with age [172,173]. In contrast, the role of metabolic stress in promoting HSC aging is still unknown.

Multiple research articles reveal that aged HSC phenotypes can be modulated by pharmacological inhibitors and genetically. Such manipulation is known to target HSC aging to restore their function and activity [122,174,175,176,177,178,179]. Cell division control protein 42 homolog (Cdc42) is a Rho GTPase that controls cell division. Cdc42 expression changes with age, leading to a loss of polarity in aged HSCs [177]. The polarization of aged HSCs and the levels and spatial distribution of histone H4K16ac were restored by inhibiting Cdc42 with an casein kinases inhibitor (CASIN) inhibitor [177]. Due to the loss of polarization aged HSCs showed reduced regenerative capacity, increased myeloid-differentiation capacity, and loss of self-renewal ability [177]. Therefore, understanding the mechanisms under which aged HSCs acquire functions like young HSCs may help in developing therapies for age-related hematological disorders [180].

A recent study has demonstrated that MMP may be utilized to detect chronologically aged HSCs that seem and operate like youthful HSCs have a high transcription rate and are associated with balanced lineage-affiliated programs [181]. Furthermore, MMP enhances the transcription, and number of HSCs in the BM niche than chronological age. Enhancing MMP increases the engraftment capability of aged HSCs and reduces myeloid-biased blood production [181]. Pharmacological modulation of MMP may modify transcriptional programs; therefore, enhancing the performance of aged HSCs. This indicates that MMP may be leveraged as a biomarker for characterizing aged HSCs [181].

Increasing mitochondrial activity may modulate the epigenetic states that are associated with aging [182]. It has been shown that mitochondria have an effect on the epigenetics of cells [182]; conversion of acetyl-CoA, influences histone acetylation and gene expression [183]. During histone modification, HSCs rely on acetyl-CoA produced by mitochondrial FAO, where acetyl-CoA help to counteract hypoacetylation and hypermethylation of histones [7]. Mitochondrial DNA (mtDNA) is epigenetically regulated by adding methylation to histones [184]. Proof-reading deficiency, caused by the accumulation of the mitochondrial DNA polymerase gamma (Polg) induces mutations in HSCs, which in turn induces early aging of HSCs in mice [29].

During cells lifecycle, cells multiply and accumulate mutations; although most mutations have little impact, a small percentage may enhance cellular survival and give a growth advantage, contributing to clonal expansion. The expression of clonal hematopoiesis of indeterminate potential (CHIP, age-related clonal hematopoiesis) is important for HSC aging. Older individuals are likely to develop a small subset of HSC clones, due to this their peripheral blood production is inordinate [185]. DNA (cytosine-5)-methyltransferase 3A (DNMT3A), ASXL transcriptional regulator 2 (ASXL2), and tet methylcytosine dioxygenase 2 (TET2) are the epigenetic modifiers mostly altered in CHIP clones. In multiple cases, CHIP is associated with an elevated risk of hematological cancers. DNMT3A, ASXL2, and TET2 control and promote metabolic activity and the differentiation of HSCs. LSCs utilize OXPHOs without affecting their ability to self-renew [186,187]. Aged HSPCs showed changes in the expression of proteins involved in DNA repair, cell proliferation, and metabolism [188].

Several glycolytic enzymes are upregulated in aging HSPCs [189], possibly contributing to CHIP formation. CHIP therapy focuses on preventing the onset and progression of hematological malignancies. Insulin-like growth factor 2 mRNA-binding protein 2 (Igf2bp2) deficient animals prevented aging-related increases in HSCs and myeloid-skewed differentiation [190].

More research is needed to determine how lysosomal degradation signaling alters HSC metabolic activity both at quiescence and in response to aging since this may have therapeutic relevance given the age-related nature of many hematological disorders.

## 10. Conclusions

Our understanding of HSC metabolism is just emerging, and the rarity of HSCs is a significant obstacle to in-depth study. HSCs need minimal mitochondrial activity to enter a quiescent or dormant state. Further study on HSC metabolism is required to understand the metabolic requirements of HSCs. In contrast, HSCs with low mitochondrial activity may function just as well without using glycolysis. Furthermore, lysosomal cargo and its influence on HSC quiescence need more study. How do lysosomal metabolic pathways activate HSCs? More studies are required to find out how lysosomal cargo accumulation affects HSC quiescence, and which metabolic pathways are needed for HSC activation.

Further thorough research is needed to identify the precise involvement of lysosomes in HSC quiescence, activation, and resting states. In-depth research is required to establish how lysosomes and mitochondria are related. However, the role of lysosomal activity and mitophagy in the maintenance or restoration of HSC quiescence remains unclear. In spite of HSCs transitioning to a quiescent state following activation, due to stress, mitochondrial and lysosomal shape, numbers, and function are maintained [13,21,22,191].

It is particularly intriguing to understand how mitochondrial remodeling owing to replicative stress affects the metabolic demands of HSCs. Additional studies are needed on the impact of lysosomal degradation and their roles on aged HSC. Multiple lines of evidence suggest that the aging process causes abnormalities in the function of HSCs, particularly in metabolism, protein synthesis, and growth signaling. More research is required to understand how lysosomal signals regulate HSC metabolic activity under steady-state and stressed situations, such as cancer and aging.
